# Influence of pelvic incidence-lumbar lordosis mismatch on surgical outcomes of short-segment transforaminal lumbar interbody fusion

**DOI:** 10.1186/s12891-015-0676-1

**Published:** 2015-08-20

**Authors:** Yasuchika Aoki, Arata Nakajima, Hiroshi Takahashi, Masato Sonobe, Fumiaki Terajima, Masahiko Saito, Kazuhisa Takahashi, Seiji Ohtori, Atsuya Watanabe, Takayuki Nakajima, Makoto Takazawa, Sumihisa Orita, Yawara Eguchi, Koichi Nakagawa

**Affiliations:** Department of Orthopaedic Surgery, Eastern Chiba Medical Center, 3-6-2 Okayamadai, Togane, Chiba 283-8686 Japan; Department of Orthopaedic Surgery, Toho University Sakura Medical Center, 564-1 Shimoshizu, Sakura, Chiba 285-8741 Japan; Department of Orthopaedic Surgery, Graduate School of Medicine, Chiba University, 1-8-1 Inohana, Chuoku, Chiba city, Chiba 260-8677 Japan; Department of General Medical Science, Graduate School of Medicine, Chiba University, 1-8-1 Inohana,Chuo-ku, Chiba city, Chiba 260-8670 Japan; Department of Orthopaedic Surgery, National Hospital Organization Shimoshizu Hospital, 934-5Shikawatashi, Yotsukaido, Chiba 284-0003 Japan

**Keywords:** Sagittal alignment, Spinopelvic, Flat-back, Pelvic incidence, Lumbar lordosis, Lumbar interbody fusion

## Abstract

**Background:**

The importance of pelvic incidence-lumbar lordosis (PI-LL: PI minus LL) mismatch is emphasized in long-segment fusion for adult spinal deformity; however, there are few studies evaluating the influence of PI-LL on surgical outcomes after short-segment fusion. In this study, we have examined the effects of PI-LL mismatch on surgical outcomes of short-segment lumbar intervertebral fusion for lumbar degenerative diseases.

**Methods:**

Patients with lumbar degenerative disease treated by short-segment (1 or 2 levels) transforaminal lumbar interbody fusion were divided into Group A (PI-LL ≤ 10°: *n =* 22) and Group B (PI-LL ≥ 11°: *n =* 30). Pre-and post-operative patient symptoms were assessed by the visual analogue scale (VAS: scores 0-100 mm; for LBP, lower-extremity pain, and lower-extremity numbness), a detailed VAS for LBP while in motion, standing, and sitting, and the Oswestry disability index (ODI). Surgical outcomes were evaluated by the Nakai score (3 = excellent to 0 = poor. Post-operative data were acquired for at least one year following surgery and were compared between the two groups. Multiple regression analyses were used to evaluate the relative influence of PI-LL on each pre-and post-operative parameter (VAS, detailed VAS and ODI) adjusted for age, sex, fusion levels, body mass index, presence of scoliosis, diabetes mellitus and depression.

**Results:**

The surgical outcomes in Group A were significantly better than those of Group B. Group A showed better post-operative VAS scores for LBP, particularly LBP while standing (11.9 vs. 25.8). The results of the multivariate analyses showed no significant correlation between PI-LL and pre-operative symptoms, but did show a significant correlation between PI-LL and the post-operative VAS score for LBP, lower extremity pain, and numbness.

**Conclusions:**

This study is the first to find that PI-LL mismatch influences post-operative residual symptoms, such as LBP, lower extremity pain and numbness. Among the three types of LBP examined in the detailed VAS, LBP while standing was most strongly related to PI-LL mismatch. The importance of maintaining spinopelvic alignment is emphasized, particularly when treating patients with adult spinal deformity using long-segment fusion surgery. However, our results indicate that surgeons should pay attention to sagittal spinopelvic alignment and avoid post-operative PI-LL mismatch even when treating patients with short-segment lumbar interbody fusion.

## Background

Transforaminal lumbar interbody fusion (TLIF) is commonly used to treat patients with lumbar degenerative disease [1]. TLIF offers the advantage of obtaining a circumferential arthrodesis through a unilateral approach with minimal retraction of neural elements. Many surgeons have reported good clinical and radiological outcomes for such lumbar interbody fusion techniques. [2–5] However, there are some patients who have post-operative residual, lower extremity pain, or numbness even without post-operative complications [2–5].

Loss of lordosis has been thought to be one of the factors influencing post-operative residual symptoms [6, 7]. In 2010, Schwab et al., reported the importance of maintaining global sagittal alignment when performing long-segment spinal fusion surgery in the treatment of adult spinal deformity [8]. Their results clearly showed that post-operative global sagittal balance is significantly correlated with post-operative residual pain and disability [8]. Radiological parameters that most highly correlate with pain and disability are sagittal vertical axis (SVA), pelvic tilt (PT), and the balance of pelvic incidence (PI) and lumbar lordosis (LL) [9]. Schwab et al. proposed radiological parameter thresholds predictive of worse clinical symptoms and poorer quality of life, and concluded that a PI-LL (PI minus LL) of 10° or less was the ideal spinopelvic alignment for reducing post-operative pain and disability [9]. Therefore, surgeons should plan corrective surgeries to acquire an adequate LL that achieves a harmonious spinopelvic alignment (PI-LL 10° or less).

In previous studies [8–10], the importance of maintaining appropriate PI-LL has been emphasized, particularly for long-segment fusion for adult spinal deformity; however, there are few studies evaluating the influence of PI-LL on surgical outcomes following short-segment fusion. In this study, we examined the effect of PI-LL mismatch on surgical outcomes of short-segment TLIF for lumbar degenerative diseases.

## Methods

Patients with lumbar degenerative diseases, such as lumbar spinal stenosis, degenerative spondylolisthesis and spondylolytic spondylolisthesis, treated by short-segment 1 or 2 level TLIF were included in the present study (Table 1). Patients who showed any indication of other pathological problems, such as vertebral fractures, tumors, or infectious diseases, were excluded. Fifty-two consecutive patients meeting the criteria were divided into Group A (PI-LL ≤ 10°: *n =* 22) and Group B (PI-LL ≥ 11°; *n =* 30) by post-operative radiological evaluation.

**Table 1:** Fusion levels and indications for fusion in groups A and B

		Group A (*n =* 22)	Group B (*n =* 30)
Number of fusion levels		1.1 ± 0.4	1.4 ± 0.5^a^
Level of fusion	(No. of cases)		
One level	L3-L4	0	1
	L4-L5	16	12
	L5-S1	4	5
Two levels	L3-L4-L5	1	9
	L4-L5-S1	1	3
Indications for fusion			
Degenerative spondylolisthesis		14	19
Spondylolytic spondylolisthesis		2	1
Spinal canal stenosis		6	10

^a^Statistically significant difference *p <* 0.05

Pre-operative characteristics of patients, including age, sex, number of fusion levels, body mass index (BMI), and incidence of degenerative scoliosis, diabetes mellitus, and depression, were compared between the two groups.

Anteroposterior and lateral radiographs in the neutral standing position were taken pre-and post-operatively. On anteroposterior radiographs, pre-operative scoliotic curvature was evaluated using the Cobb method. On lateral radiographs, PI and LL were evaluated one-year after surgery. The PI is measured as the angle between a line drawn perpendicular to the sacral end plate at its midpoint and a line drawn from the midpoint of the sacral end plate to the midpoint of the femoral head axis. The LL is the sagittal Cobb angle measured between the superior end plate of L1 and the superior end plate of S1 [8]. Post-operative parameters, such as PI, LL, and PI-LL, were then compared between the two groups. Excellent inter- and intra-rater reliability has been reported for these measurements [10].

### Evaluation of surgical outcomes and clinical symptoms

Intra-and post-operative complications were retrospectively reviewed for each group. Surgical outcomes were evaluated using the Nakai score (Table 2: 3 = excellent to 0 = poor) [11]. Pre-and post-operative patient symptoms were also assessed by the visual analogue scale (VAS: scores 0-100 mm; for LBP, lower-extremity pain, and lower-extremity numbness) and the Oswestry disability index (ODI). In addition, the detailed VAS scoring system, originally developed by us for LBP while in motion, standing, and sitting, was used for a more detailed evaluation of LBP, pre-and post-operatively [12]. Post-operative data were prospectively acquired for at least one year following surgery. All scores were compared between the two groups.

**Table 2:** Nakai score

Scoring used in the study	
The patient has resumed work-related and other activities with slight or no symptoms.	3: Excellent
The patient has resumed work-related and other activities but occasionally feels pain in the back or lower limbs after strenuous work.	2: Good
The patient has reduced work-related and other activities because of residual pain in the back or lower limbs.	1: Fair
The patient cannot work or carry out activities of daily living and is considered to be disabled.	0: Poor

### Surgical procedures

All patients underwent surgery in the prone position after induction of general endotracheal anesthesia. The side selected for the unilateral approach was the side showing more neurological involvement. If symptoms were equal on both sides, the approach was made on the left side. A midline incision exposed the posterior elements and the lateral aspect of the facet joint. Following ipsilateral laminotomy, partial facetectomy exposed the surface of the intervertebral disc. Resection of the bone was performed manually using an osteotome and Kerrison rongeur to preserve local bone. Following the removal of disc material and meticulous endplate preparation, the resected local bone was milled and packed into the intervertebral disc space as a bone graft. One or two fusion cages packed with milled local bone were then inserted into the disc space. Contralateral decompression was performed if needed; the contralateral ligamentum flavum, ventral aspect of the lamina, and medial aspect of the contralateral facet joints were resected through the ipsilateral side. Following neural decompression, pedicle screws were placed on both sides. A 1-cm fascia incision allowed insertion of pedicle screws transfascially, using a percutaneous pedicle screw system. Adequate compressive force by the pedicle screws was applied to the disc space to establish stability of the fusion cages.

The percutaneus pedicle screws used for the patients in this series were Viper (Depuy Synthes Spine, Raynham, MA) or Mantis (Stryker, Kalamazoo, MI).

Written informed consent was obtained prior to surgery. The study protocol was approved by the institutional ethics committee of Toho University Sakura Medical Center (No. 2012–071).

### Statistical analysis

Statistical analyses used were the unpaired t-test for age, fusion levels, BMI, PI, LL, PI-LL and VAS, the Mann-Whitney U test for the Nakai score and ODI, and the chi-square test for sex and incidence of each disease.

Multiple regression analysis was performed to investigate the relative influences of pre-and post-operative PI-LL on each pre-and post-operative VAS, detailed VAS, and ODI after adjustment for age, sex, fusion levels, BMI, presence of scoliosis, diabetes mellitus and depression. PI-LL was utilized as the independent variable and pre-and post-operative data for VAS and ODI as the dependent variables.

Probability values of *p <* 0.05 were considered significant. Values are expressed as the mean ± standard deviation (SD).

## Results

Patient characteristics of Group A (mean age: 67.9 years; range: 44–83 years: 14 male and 8 female) and Group B (mean age: 65.6 years; range: 35–86 years: 11 male and 19 female), are presented in Table 3. No significant differences were observed between the two groups, except in the post-operative radiological evaluations. Significant differences between groups were found in post-operative PI, LL, and PI-LL (Table 3). The post-operative LL (Group A: 44.0 ± 8.3; Group B: 29.4 ± 10.7) was not significantly different from the pre-operative LL (Group A: 43.1 ± 11.4; Group B: 30.4 ± 12.4) in both groups. As described in Table 1, the number of fusion levels differed significantly between the two groups.

**Table 3:** Characteristics of patients in groups A and B

		Group A (≤10 deg.; *n =* 22)	Group B (≥11 deg.; *n =* 30)	*p* value
Age	(years)	67.9 ± 8.3	67.5 ± 11.8	NS
Sex	(M/F)	14/8	11/19	NS
Body mass index	(kg/m^2^)	23.7 ± 2.6	25.4 ± 3.9	NS
Degenerative scoliosis	(≥10 deg)	22.7 % (5/22)	40.0 % (12/30)	NS
Diabetes mellitus		0.0 % (0/22)	10.0 % (3/30)	NS
Depression		4.5 % (1/22)	6.7 % (2/30)	NS
Pelvic incidence (PI)^a^	(Degree)	44.9 ± 7.0	52.1 ± 11.7	*p* = 0.013
Lumbar lordosis (LL)^a^	(Degree)	44.0 ± 8.3	29.4 ± 10.7	*p* < 0.001
PI minus LL (PI-LL)^a^	(Degree)	1.0 ± 5.8	22.7 ± 8.9	*p* < 0.001
Follow-up	(months)	16.9 ± 6.6	15.9 ± 5.3	NS

^a^postoperative data; NS is non-significant at *p >* 0.05

### Surgical complications

In Group A, one patient had a pulmonary embolism, but recovered with anticoagulant therapy, and one patient had a post-operative epidural hematoma that required additional surgery (Table 4). In Group B, one patient had post-operative cage migration, one patient had pedicle screw malposition that required corrective surgery and two patients had dural tears that required dural sutures (Table 4). The incidence of surgical complication did not differ significantly between the two groups. No long-term negative effects continued until the final follow-up in any case with a surgical complication. Homologous blood transfusions were not required in either group.

**Table 4:** Complications during the 1-year minimum follow-up

	Group A no. of cases (%)	Group B no. of cases (%)
Cage migration	0 (0.0)	1 (3.3)
Screw malposition	0 (0.0)	1^a^ (3.3)
Pulmonary embolism	1 (4.5)	0 (0.0)
Dural tear	0 (0.0)	2^b^ (6.6)
Infection	0 (0.0)	0 (0.0)
Postoperative epidural hematoma	1^a^ (4.5)	0 (0.0)
Blood transfusion	0 (0.0)	0 (0.0)
Re-operation	1 (4.5)	1 (3.3)

^a^Cases requiring additional surgery to correct complications. ^b^Dural sutures required

### Comparisons of the Nakai score, pre-and post-operative VAS scores, and ODI

Surgical outcomes and post-operative residual symptoms evaluated at 16.9 ± 6.6 (12–32) months in Group A, and 15.9 ± 5.3 (12–29) months in Group B, did not differ significantly between the two groups. In Group A, all 22 patients had good or excellent surgical outcomes, compared to 80.0 % (24/30) in Group B. The Nakai scores converted to 0 = poor to 3 = excellent were compared between the two groups. Group A exhibited a significantly better score than Group B (*p* = 0.008) (Table 5).

**Table 5:** Comparisons of pre-and post-operative scores for the visual analogue scale (VAS), Oswestry disability index (ODI), and detailed VAS for low back pain while in motion, standing, and sitting in groups A and B

		Group A	Group B	*p* value
Nakai score		2.59 ± 0.50	2.07 ± 0.78	*p* = 0.008
Low back pain (LBP)	Pre-op	48.9 ± 27.5	63.2 ± 22.6	*p* = 0.046
	Post-op	14.1 ± 19.4	23.8 ± 20.9	NS (*p* = 0.096)
Lower extremity pain	Pre-op	68.2 ± 24.8	66.9 ± 25.3	NS
	Post-op	13.7 ± 19.2	20.9 ± 23.3	NS
Lower extremity numbness	Pre-op	62.8 ± 27.4	64.3 ± 32.0	NS
	Post-op	11.7 ± 17.3	20.2 ± 26.7	NS
ODI	Pre-op	41.3 ± 15.4	47.4 ± 18.7	NS
	Post-op	17.1 ± 13.1	22.0 ± 15.7	NS
LBP while in motion	Pre-op	49.1 ± 35.2	55.6 ± 32.2	NS
	Post-op	19.2 ± 26.0	22.6 ± 23.9	NS
LBP while standing	Pre-op	63.8 ± 30.9	73.0 ± 25.0	NS
	Post-op	11.9 ± 17.6	25.8 ± 26.7	*p* = 0.039
LBP while sitting	Pre-op	37.0 ± 27.4	43.9 ± 27.2	NS
	Post-op	12.3 ± 17.3	16.1 ± 19.4	NS

Nakai score: 3 = excellent to 0 = poor; VAS scores: 0–100 mm

Values are expressed as the mean ± standard deviation. NS is non-significant at *p >* 0.05

In VAS scores for LBP, Group A showed a better score, both pre- and post-operatively, with a significant difference pre-operatively (*p* = 0.046), and a non-significant tendency post-operatively (*p* = 0.096) (Table 5). No significant difference was found in pre-and post-operative VAS scores for lower extremity pain and numbness between the two groups (Table 5).

The ODI showed no significant differences between the two groups pre-and post-operatively (Table 5).

A more thorough analysis of LBP using the detailed VAS scoring system revealed that both groups showed similar pre-operative levels of LBP while standing, but post-operatively, Group A showed significantly less LBP while standing than Group B (*p* = 0.039) (Table 5). In both pre-and post-operative data, no significant differences were found in LBP while in motion and while sitting between the two groups (Table 5).

### Correlation between PI-LL and post-operative symptoms

A multivariate analysis was performed, adjusted for age, sex, fusion levels, BMI, presence of scoliosis, diabetes mellitus and depression, with pre-and post-operative VAS and ODI as dependent variables, and PI-LL as an independent variable. The results showed no significant association between PI-LL and pre-operative parameters (Table 6). However, greater PI-LL was significantly associated with worse post-operative VAS for LBP (*p* = 0.045), lower extremity pain (*p* = 0.024), and numbness (*p* = 0.019) (Table 7). No significant association was found between PI-LL and post-operative ODI (Table 7). Among the detailed VAS scores in the three situations, a larger PI-LL was significantly associated with worse LBP while standing (*p* = 0.038), but there was no significant association with LBP while in motion or sitting (Table 7).

**Table 6:** Correlations between PI-LL and pre-operative VAS, detailed VAS, and ODI

Valuables	Regression coefficient	Standardized regression coefficient	t value	*p* value
VAS for LBP	0.355	0.198	1.216	*NS*
				*p* = 0.229
VAS for lower	0.390	0.223	1.377	*NS*
Extremity pain				*p* = 0.174
VAS for lower	0.078	0.037	0.235	*NS*
Extremity numbness				*p* = 0.815
ODI	0.382	0.310	1.980	*NS*
				*p* = 0.053
Detailed VAS for	0.371	0.158	0.938	*NS*
LBP while in motion				*p* = 0.353
Detailed VAS for	0.384	0.197	1.186	*NS*
LBP while standing				*p* = 0.241
Detailed VAS for	0.474	0.248	1.466	*NS*
LBP while sitting				*p* = 0.149

NS is non-significant at *p >* 0.05

*PI* pelvic incidence; *LL* lumbar lordosis; *PI-LL* PI minus LL; *VAS* visual analogue scale; *ODI* Oswestry disability index; *LBP* low back pain

**Table 7:** Correlations between PI-LL and post-operative VAS, detailed VAS, and ODI

Valuables	Regression coefficient	Standardized regression coefficient	t value	*p* value
VAS for LBP	0.517	0.335	2.052	*p* = 0.045
VAS for lower extremity pain	0.577	0.348	2.321	*p* = 0.024
VAS for lower extremity numbness	0.639	0.360	2.417	*p* = 0.019
ODI	0.241	0.215	1.372	*NS*
				*p* = 0.176
Detailed VAS for	0.401	0.213	1.288	*NS*
LBP while in motion				*p* = 0.204
Detailed VAS for LBP while standing	0.626	0.341	2.134	*p* = 0.038
Detailed VAS for	0.379	0.270	1.632	*NS*
LBP while sitting				*p* = 0.109

NS is non-significant at *p >* 0.05

*PI* pelvic incidence; *LL* lumbar lordosis; *PI-LL* PI minus LL; *VAS* visual analogue scale; *ODI* Oswestry disability index; *LBP* low back pain

## Discussion

Post-operative flat-back syndrome has been reported to be a factor causing undesirable surgical outcomes after lumbar fusion surgery [6, 7]. Schwab et al. recently proposed a systematic evaluation method to classify sagittal spinal alignment in flat-back cases [8]. Their study demonstrated that SVA, PT, and PI-LL were most closely related to LBP and disability. Since the Scoliosis Research Society-Schwab adult spinal deformity classification was established [10], there have been no studies examining the influence of PI-LL mismatch on surgical outcomes after short-segment lumbar interbody fusion. Our study is the first to show that PI-LL mismatch significantly correlated with surgical outcomes after short-segment lumbar interbody fusion. Consequently, our study reveals that surgical outcomes after short-segment lumbar interbody fusion will be poorer when good spinopelvic balance is not achieved following surgery. Thus, surgeons should pay attention to the sagittal spinopelvic balance of patients even when treating patients with short-segment lumbar spinal stenosis or spondylolisthesis. Our results show that post-operative PI-LL mismatch causes greater residual LBP. Interestingly, in this study, our originally developed detailed VAS system was successful in revealing that LBP while standing is most closely correlated with post-operative PI-LL mismatch. Patients with flat-back syndrome are usually characterized by an inability to stand erect and back pain [7]. In our study, post-operative residual LBP while standing may reflect the inability to stand erect due to flat-back syndrome.

We believe that PI-LL did not correlate with pre-operative symptoms (Table 6) because pre-operative symptoms, including pain caused by nerve root compression, spinal instability, and spinopelvic alignment, are more complex than post-operative symptoms. Post-operative symptoms, particularly LBP while standing, were more simply influenced by spinopelvic alignment (Table 7), because symptoms caused by nerve root compression and spinal instability may have been relieved by fusion surgery. Therefore, PI-LL significantly correlated with post-operative LBP, but not with pre-operative LBP (Tables 6, 7).

There are some limitations to this study. Most importantly, post-operative residual symptoms are affected by multiple factors. In our study, Group A showed patient characteristics that differed from those of Group B. Although most were not significant (Tables 1, 3), the possibility that differing characteristics influenced the results of the comparison study between Group A and Group B persists. In particular, the difference in number of fusion levels may affect surgical outcomes because better surgical outcomes would be expected from single-level fusion than two-level fusion [13]. However, the multivariate analysis adjusted for age, sex, fusion levels, BMI, presence of scoliosis, diabetes mellitus and depression showed that PI-LL was significantly associated with post-operative symptoms, not with pre-operative symptoms. These results lead us to conclude that post-operative PI-LL mismatch causes more intense post-operative residual symptoms, particularly LBP while standing. There are other limitations, including the short post-operative follow-up and small number of patients, and the variety of pathologies that were indicators for fusion surgery in the study. There is a concern that short-term outcomes may be influenced by surgery-related factors, rather than radiologic factors. However, in our study no patients with surgical complication had deleterious consequences from the complication after additional treatments, such as screw reposition, hematoma removal, or dural suture were accomplished. Recent studies reported that a post-operative PI-LL mismatch resulted in increased load on adjacent segments and predisposition to adjacent segment pathologies and late surgical complications seen after longer follow-ups [14–17]. Thus, longer follow-up times may reveal an effect of adjacent segment pathology or late surgical complications on surgical outcomes. However, the statistically significant differences found between our two study groups, despite the small number of subjects, lead us to believe our results are reliable and provide important information for spine surgeons treating lumbar degenerative disease. Further investigations in which inclusion criteria are stricter with a longer follow-up and larger number of subjects are needed to confirm our conclusions.

In this study, we did not examine the effects of correcting LL on surgical outcomes because the surgery in this series was not intended to correct LL. Therefore, we cannot conclude that the surgical correction of LL would have a favorable effect on surgical outcomes. However, there have been several studies showing the positive effects of improving sagittal spinopelvic alignment on surgical outcomes of short-segment fusion surgery. Kim et al. found that patients with improved PT after posterior lumbar interbody fusion (PLIF) had better clinical results [18]. Lazennec et al. reported that a larger post-operative PT positively correlated with LBP after lumbosacral fusion surgery [19]. A larger PT, which reflects pelvic retroversion, is usually correlated with loss of LL [20]; therefore, we surmise that the surgical correction of LL decreases PT and produces improved surgical outcomes. Hioki et al. found that two-level PLIF provided satisfactory results by preserving lordosis of the lumbar spine, suggesting the importance of restoring LL by fusion surgery [21]. However, Lazennec et al. reported that post-operative loss of lordosis was not always related to post-operative residual symptoms [19]. One explanation for this inconsistency may be that the appropriate LL varies depending on a patient’s PI. These findings indicate the importance of acquiring ideal PI-LL when performing short-segment lumbar fusion.

More recently, Kim et al. reported that TLIF has the potential to cause post-operative flat back, and that PLIF is more reliable than TLIF for restoring spinopelvic alignment [22]. For the maintenance of segmental lumbar lordosis, extreme lateral interbody fusion (XLIF) is thought to have more capability to increase disc height and correct spinopelvic alignment [23–25]. From these observations, surgeons can select the appropriate surgical technique from the various fusion methods, TLIF, PLIF, XLIF, multi-segment fusion, spinal osteotomy, etc., taking into consideration the ability of each method to restore spinopelvic alignment. Efforts should be made to reduce PI-LL to 10° or less whenever feasible.

## Conclusions

This study is the first to report that PI-LL influences post-operative residual symptoms after short-segment fusion for lumbar degenerative disease. PI-LL mismatch is more closely related to LBP while standing than LBP while in motion or sitting. From these observations, surgeons should be careful to achieve sagittal spinopelvic alignment and avoid post-operative PI-LL mismatch when treating patients with short-segment lumbar interbody fusion, particularly when the patient has severe LBP while standing.

## Abbreviations

PI: Pelvic incidence
LL: Lumbar lordosis
VAS: Visual analog scale
ODI: Oswestry disability index
LBP: Low back pain
TLIF: Transforaminal lumbar interbody fusion
SVA: Sagittal vertical axis
PT: Pelvic tilt
BMI: Body mass index
PLIF: Posterior lumbar interbody fusion
XLIF: Extreme lateral interbody fusion
